# What Have We Learned about the Microbiomes of Indoor Environments?

**DOI:** 10.1128/mSystems.00083-16

**Published:** 2016-07-26

**Authors:** Brent Stephens

**Affiliations:** Department of Civil, Architectural and Environmental Engineering, Illinois Institute of Technology, Chicago, Illinois, USA; MIT

**Keywords:** buildings, indoor air quality, indoor environment, indoor microbiomes

## Abstract

The advent and application of high-throughput molecular techniques for analyzing microbial communities in the indoor environment have led to illuminating findings and are beginning to change the way we think about human health in relation to the built environment. Here I review recent studies on the microbiology of the built environment, organize their findings into 12 major thematic categories, and comment on how these studies have or have not advanced knowledge in each area beyond what we already knew from over 100 years of applying culture-based methods to building samples. I propose that while we have added tremendous complexity to the rich existing knowledge base, the practical implications of this added complexity remain somewhat elusive. It remains to be seen how this new knowledge base will change how we design, build, and operate buildings. Much more research is needed to better understand the complexity with which indoor microbiomes may affect human health in both positive and negative ways.

## Abstract (precis)

The advent and application of high-throughput molecular techniques for analyzing microbial communities in the indoor environment have led to illuminating findings and are beginning to change the way we think about human health in relation to the built environment. Here I review recent studies on the microbiology of the built environment, organize their findings into 12 major thematic categories, and comment on how these studies have or have not advanced knowledge in each area beyond what we already knew from over 100 years of applying culture-based methods to building samples.

## INTRODUCTION

Over the last decade, there has been a dramatic increase in the use of high-throughput molecular techniques to analyze microbial communities in indoor environments. The U.S. National Academies of Sciences, Engineering, and Medicine (NAS) is now conducting a consensus study to “examine the formation and function of microbial communities in built environments, the impacts of such microbial communities on human health, and how human occupants shape complex indoor microbiomes” (http://nas-sites.org/builtmicrobiome/). The NAS study is cosponsored by the Alfred P. Sloan Foundation, U.S. Environmental Protection Agency (EPA), National Aeronautics and Space Administration (NASA), and National Institutes of Health (NIH). To help the NAS committee understand the current state of research in this area, I recently presented a talk entitled “Perspectives on microbial interactions in built environments,” in which I did the following: (i) reviewed recent studies on the microbiology of the built environment, (ii) organized their findings into 12 major thematic categories, (iii) proposed that we have added many new layers of complexity to the rich existing knowledge base from a long history of applying culture-based methods to analyze microbes in indoor environments, and (iv) proposed that the practical implications of this added complexity remain somewhat elusive. Here I summarize my presentation and findings, with some minor modifications. (My presentation can be downloaded at https://dx.doi.org/10.6084/m9.figshare.3459257.v1.)

## TWELVE LESSONS THAT WE HAVE LEARNED ABOUT THE MICROBIOMES OF INDOOR ENVIRONMENTS

### (i) Culture-independent methods reveal vastly greater microbial diversity compared to culture-based methods.

Over the last decade, there has been a dramatic increase in the use of high-throughput molecular techniques to analyze microbial communities in indoor environments (1–3). The advent and application of culture-independent molecular methods for analyzing microbial communities (e.g., 16S and internal transcribed spacer [ITS] rRNA sequencing, shotgun metagenomics, and quantitative PCR [qPCR]) have revealed vastly greater microbial diversity present in environmental samples compared to traditional culture- and microscopy-based methods (typically on the order of ~100:1) (4–6). While applications of culture-based methods had previously provided excellent insight into the quantity and types of microbes found indoors, methods were limited to quantification of only the viable (and culturable) microbes in air and surface samples inside buildings, with some level of identification for well-characterized species or genera based on physical characteristics.

For example, Tsai and Macher found that indoor air in 100 U.S. office buildings contained a smaller quantity of culturable bacteria than what was found outdoors and that indoor air included a combination of mostly Gram-positive cocci and rods (7). Conversely, Moschandreas et al. found that indoor air in 20 residences in Chicago, IL, contained greater amounts of culturable bacteria than outdoor air, and that *Staphylococcus* species (which are well-known to be ubiquitous on human skin [8]) made up nearly one-third of the indoor culturable bacteria (9). However, more-recent studies utilizing molecular methods have illuminated “an entirely new dimension of microbial diversity” in indoor environments ranging from child-care facilities (10) to residences (11). As an example of this vastly increased complexity, a recent study found that the *Staphylococcus* genus comprised only ~4% of the identifiable taxa in indoor air samples from 29 homes in San Francisco, CA, with major contributions from nearly 20 other taxa ranging from *Comamonadaceae* to *Methylocystaceae* (12).

It is also worth noting two things here before moving forward. (i) To date, the vast majority of indoor microbiome investigations have analyzed bacterial communities using 16S sequencing and/or fungal communities using ITS sequencing, with much less being known about viral communities found in indoor environments (13–15). (ii) Accurate bacterial or fungal community identification with short-read sequencing (which represents the majority of indoor microbiome studies thus far) typically yields results only at the family or genus level.

### (ii) Indoor spaces often harbor unique microbial communities.

The application of next-generation sequencing and advanced statistical analysis techniques has demonstrated that indoor spaces often harbor unique microbial communities in ways that we did not previously understand. For example, an early study of settled dust in two buildings in Finland found that there were clear differences in bacterial flora in each building and that the differences between buildings were greater than the differences between seasons (16). Hewitt et al. found that bacterial communities on surfaces in offices in Tucson, AZ, were clearly distinguishable from those in New York, NY, and San Francisco, CA, while bacterial counts were higher on surfaces in Tucson and New York than in San Francisco (17). More recently, Lax et al. found that microbial communities on surfaces in several U.S. residences differed substantially among homes and that microbiota in each home were identifiable by family (11). Further, Meadow et al. found that people release their own personalized microbial cloud with distinct microbial communities that can be used to identify individual occupants (18).

### (iii) Indoor fungal communities are largely driven by outdoor fungal communities in nondamp buildings.

In buildings without prior moisture and dampness problems, outdoor fungal communities largely drive indoor fungal communities. For example, Amend et al. demonstrated that fungal diversity in settled-dust samples from 72 buildings across the world was higher further from the equator and that building function had no significant effect on indoor fungal composition, despite stark differences in building designs and materials (19). The same has been recently confirmed in house dust samples from approximately 1,200 U.S. homes (20). Similarly, albeit on a more-local scale, Adams et al. found that indoor fungal communities were strongly influenced by dispersal from outdoors and that there were no fungal taxa found as indicators of indoor sources (21).

### (iv) Indoor fungal communities in damp buildings are often distinct from those in nondamp buildings.

In buildings with prior moisture and dampness problems, indoor fungal communities are often distinct from those in nondamp buildings. A recent example of this phenomenon is shown by Emerson et al., where the application of qPCR to indoor air samples in Boulder, CO, demonstrated that fungal abundances were approximately three times higher in flood-damaged homes compared to nonflooded homes and that *Penicillium* was the most abundant taxon in flooded homes (22). However, it is also worth noting that the use of culture-based methods in flooded versus nonflooded buildings had also revealed similar findings at least 15 years prior (23–26).

### (v) Indoor bacterial communities often originate from indoor sources.

In an early study using molecular methods, Tringe et al. found that although indoor air was much less diverse than other environments traditionally studied by microbiologists (e.g., soil and water), indoor microbes appear to mostly originate from indoor niches (27). Several other studies have shown this to be particularly true of indoor bacterial communities (11, 28–33), although I will save more-detailed descriptions of some of these studies for subsequent sections.

### (vi) Source-tracking techniques demonstrate that humans and pets often dominate bacterial communities on indoor surfaces.

Repeated studies of varied indoor environments have used source-tracking algorithms (34) to illustrate that humans (and pets, if present) often dominate bacterial communities found on indoor surfaces (20). Microbes associated with human skin (and to a lesser extent, the human gut) have been shown to be ubiquitous on surfaces in a wide variety of buildings, including public restrooms (15, 29), residential kitchens (28), neonatal intensive care units (35), and bathrooms, bedrooms, and other commonly occupied microenvironments in homes (30). Source-tracking techniques have also been used to reveal how changes in human occupancy affect microbes on surfaces. For example, Lax et al. demonstrated that when a person traveled away from home for a few days, the relative contribution of bacterial taxa associated with that person rapidly declined but then rapidly increased on many surfaces after the person returned home (11). Further, after a family moved into a new house, the microbial community in the new house rapidly converged on the microbial communities found in the occupants’ former house. As another example, Gibbons et al. demonstrated that gut- and skin-associated taxa persisted for weeks to months on surfaces in a public restroom after initial dispersal from humans (15).

### (vii) Occupants and surfaces interact in both directions.

Building on the high-time-resolution data in Lax et al. (11), unpublished analysis of microbial communities on surfaces in a new hospital before and after it became occupied with patients and staff (http://hospitalmicrobiome.com) revealed that occupants and surfaces can interact in both directions with regard to their associated microbes. More specifically, Lax et al. found that bacterial communities sampled from the hands of patients in the hospital became more similar to the bacterial communities sampled from the floor of their rooms with each additional day of stay (S. Lax, N. Sangwan, D. Smith, P. Larsen, K. Handley, M. Richardson, E. Landon, J. A. Siegel, J. C. Alverdy, R. Knight, B. Stephens, and J. A. Gilbert, submitted for publication). However, taxa shared with the skin of the current patient were found to be more abundant on patient room surfaces after the patient had spent a night in the room, while taxa shared with room surfaces were more abundant on the patient’s skin when the patient first entered a new patient room. In other words, patients appear to initially acquire room-associated bacterial taxa that predate their stay, but their own microbial signatures later influence the room (and longer stays further encourage the latter).

### (viii) Humans are also major sources of bacteria to indoor air.

In addition to being major sources of bacteria on surfaces, a number of studies have demonstrated that humans are also major sources of bacteria to indoor air. For example, Hospodsky et al. found that human occupancy in a university classroom increased the total bacterial genome concentration in indoor air nearly 2 orders of magnitude compared to unoccupied periods (31). Hospodsky et al. also demonstrated that human emissions were the dominant source of bacterial concentrations measured in five of six occupied children’s classrooms that they studied and that outdoor air ventilation was the dominant bacterial source in only one of those classrooms (36). Qian et al. estimated that students in a university classroom emit ~37 × 10^6^ bacterial genomes per person per hour and ~7.3 × 10^6^ fungal genomes per person per hour, on average (37). Subsequent real-time measurements made using an UV aerodynamic particle sizer (UV-APS) further demonstrated that classroom occupants emit ~2 × 10^6^ fluorescent biological aerosol particles (FBAPs) per person per hour, on average, and that more vigorous occupant activity during transitions between lectures led to greater FBAP emissions.

### (ix) Controlled studies can elucidate the mechanisms of human microbial emissions.

Subsequently, more-controlled chamber studies have elucidated the relative importance of three main mechanisms of human microbial emissions: (i) direct shedding from skin and clothing, (ii) resuspension of settled particles, and (iii) direct surface contact. Bhangar et al. used a UV-APS to measure microbial emissions from people seated in a chamber doing simulated office work and found an average value of ~10^6^ FBAPs per person per hour (38). Walking increased FBAP emissions approximately 5 to 6 times compared to seated office work. Further, during both walking and sitting, more than two-thirds of the emissions were found to originate from the floor (i.e., resuspension was the dominant mode), while direct shedding from skin and clothing contributed to the remaining emissions. The dominant particle size was ~3 to 5 µm for all activities.

### (x) Building design and operation can influence indoor microbial communities.

Several studies have shown that building design and operation can influence indoor microbial communities. As an example of the impact of building operation, the source (and delivery rate) of outdoor ventilation air has been shown to have a large influence on indoor microbial communities. Kembel et al. found that when a room was ventilated primarily via outdoor airflow through an open window, it had a higher level of bacterial diversity than when the room was ventilated using a mechanical heating, ventilation, and air-conditioning (HVAC) system with the window closed (39). Kembel et al. found similar results in offices, where the source of ventilation air had the greatest effect on bacterial community structure (40). Their results clearly demonstrated that the relative abundance of certain taxa were more prevalent during window ventilation periods than mechanical ventilation periods (and vice versa). Further, in buildings with high outdoor air ventilation rates, indoor air bacterial communities tend to more closely track those found in outdoor air, which diminishes the influence of human emissions more so than in buildings with lower outdoor air ventilation rates (41, 42). As another set of examples of the influence of building operation, Dannemiller et al. found that higher fungal richness (i.e., the mean number of fungal operational taxonomic units [OTUs]) was associated with a higher prevalence of air-conditioner use in homes (43), and Weikl et al. found that variations in fungal communities in house dust were significantly correlated with the prevalence of window opening (44).

As an example of the impact of building design, Kembel et al. also found that spaces with a high human occupant diversity and a high degree of physical connectedness to other spaces contained a unique collection of bacterial taxa compared to spaces with low levels of connectedness and occupant diversity (40). As another example of the influence of design, Weikl et al. found that variations in fungal communities in house dust were explained in part by the surrounding greenness of adjacent outdoor spaces, as well as the age of the buildings (44).

### (xi) Building environmental conditions often have a small influence on indoor microbial communities.

Although building design and operation have been shown to have a large impact on indoor microbial communities, building environmental conditions have typically revealed small or negligible associations with microbial community measures. I define the term building environmental conditions here as concurrent measurements of physical parameters, such as indoor air or surface temperature, relative humidity or absolute humidity, illuminance, occupancy, and others (45). For example, in the aforementioned study of a new hospital before and after it officially opened, my research group made long-term high-resolution measurements of built environment parameters, including the temperature, relative humidity, humidity ratio of indoor air, illuminance, room occupancy (measured via doorway beam breaks), CO_2_ concentrations, room pressurization, and outdoor air fractions in the HVAC systems serving the sampled spaces (46, 47). Only temperature, humidity, and illuminance were found to have statistically significant (albeit very small) impacts on microbial similarity between patients and room surfaces (Lax et al., submitted).

Another recent study assessed microbial composition on three common types of surface materials (i.e., ceiling tile, carpet, and drywall) located in three locations (i.e., on the floor, wall, and ceiling) in offices in three U.S. cities, alongside a number of built environment parameters, including equilibrium relative humidity at the material surface, room occupancy, temperature, relative humidity, and illuminance (48). Measures of bacterial community composition were not found to be associated with any of the indoor or material environmental parameters that were assessed, while fungal community richness was only weakly correlated with equilibrium relative humidity measured at the surface of the building materials. This work adds to the evidence (15) that most indoor environments (without prior water or dampness problems) are extremely scarce in water and nutrients and that although microbes are clearly dispersed onto surfaces, they likely either die or lie dormant, “waiting for liquid water to become active again” (49). As another example of the importance of liquid water, Dannemiller et al. found that the presence of water leaks was associated with greater fungal richness in house dust (43).

It is also worth noting that some of the measures of microbial communities made using modern molecular techniques may not be particularly useful for comparing to concurrent building environmental conditions. For example, measures of occupancy are clearly expected to influence the quantity of bacteria in indoor air in most indoor environments (31, 36-38, 50), and it is well-known that environmental conditions affect microorganism survival on surfaces and in air (51-63). However, plausible mechanisms for how or why environmental conditions would be expected to influence measures such as microbial richness, diversity, similarity to other samples, or the relative abundance of particular taxa in a sample remain unclear.

### (xii) Exposures to the “right” number of the “right” kinds of microbes may be beneficial for human health.

Last, after decades of indoor microbiology research focusing primarily on human exposure to infectious agents (64-67) and asthma/allergy triggers (68-70) that yield adverse health outcomes, evidence is emerging that some measures of microbial diversity and/or abundance in indoor environments may actually be beneficial for human health. For example, Fujimura et al. demonstrated that mice exposed to dog-associated house dust had a distinct gut microbiome composition (i.e., enriched for *Lactobacillus johnsonii*) and were protected against airway allergen challenges (71). Dannemiller et al. found that in human populations, lower fungal diversity in house dust (assessed by number of fungal OTUs) was significantly associated with childhood asthma development in a small case-control study (72). Further, a decrease in the genus *Cryptococcus* was significantly associated with increased asthma risk, while no fungal taxa were positively associated with asthma development. In a larger birth cohort, Lynch et al. found that while cumulative residential allergen exposure over the first 3 years of life was associated with allergic sensitization at age 3, first-year exposure to some allergens (e.g., cockroach, mouse, and cat allergens), as well as reduced exposure to specific *Firmicutes* and *Bacteriodetes*, were associated with atopy and atopic wheeze (73). Thus, exposure to high levels of certain allergens and certain bacteria in very early life stages might be beneficial for health. Somewhat similarly, Dannemiller et al. found that increased concentrations of the sum of allergenic fungal species and total fungal concentrations were both associated with increased asthma severity in a cohort study of asthmatic children (74). Conversely, some genera, including the yeast genus *Kondoa*, appeared to be protective against asthma severity. While these studies remain limited, they offer promising insight into the complexity of the impacts that indoor microbiomes may have on human health.

## WHAT ARE THE PRACTICAL IMPLICATIONS OF THIS RECENT WORK?

The advent and application of molecular sequencing techniques for investigating microbes in the indoor environment have led to illuminating findings and are beginning to change the way we think about human health in relation to the built environment. However, I propose that while we have added tremendous complexity to the rich existing knowledge base from a long history of applying culture-based methods to analyze microbes in indoor environments, the practical implications of this added complexity remain somewhat elusive. The point is probably best illustrated by one of the oldest published investigations of indoor air of which I am aware, an article published in 1887 by Carnelley et al. entitled “The Carbonic Acid, Organic Matter, and Micro-Organisms in Air, More Especially of Dwellings and Schools” (75). Konya and Scott originally drew my attention to this paper (2), which I describe in more detail here with several direct quotations.

In this wide-ranging work that was well ahead of its time, Carnelley et al. (75) investigated, among other things, “the sources of the organic matter and micro-organisms of air inside buildings, and the circumstances affecting the number of micro-organisms; also of the relative number of bacteria and moulds in b oth outside and inside air.” They conducted measurements inside and outside a large number of homes and schools in Scotland. They collected air samples by drawing air through a glass tube lined with “meat jelly” to collect viable microbes for subsequent quantification. I encourage a thorough reading of this extremely detailed work, but here I summarize some of their most important findings as they relate to research on microbes in the built environment (Table 1).

**TABLE 1:** Practical implications of much of this work have not advanced very far beyond 1887

Direct quote(s) from Carnelley et al. (75)	Translation and/or comment (if applicable)
“In order to draw conclusions from an examination of air inside buildings, it is of course necessary to know the state of the outside air.”	Always sample both indoor and outdoor air. Most, but not all, studies reviewed in the previous sections followed this advice.
“The explanation of the ratio Bacteria:Moulds increasing with the vitiation of the air is that moulds come mostly from the outside air. When the air in a room becomes vitiated the bacteria increase largely, while the number of moulds is affected to a relatively much less extent, if at all.”“when a room is left quiet the micro-organisms settle out in a few hours, so that the air becomes comparatively free.”	Fungi mostly come from outdoors, while bacteria are mostly emitted indoors. This is mostly consistent with findings iii, v, vi, and viii above.
“Hence it is clear that a certain amount of physical disturbance in a room is a condition necessary to the presence of micro-organisms in the air.”“The skin and clothes of the persons present in a room at the time of an observation also occur naturally as a probable source of infection of air.”	Resuspension, as well as direct shedding from skin and clothes, are two main sources of indoor microbes. This is consistent with finding ix above. I should note that this study also found that the “cleanliness of rooms and persons habitually in them” affected airborne microbe counts. Higher “micro-organism counts” were found in homes that were classified as “very dirty” compared to “clean” upon visual inspection of both the house and its occupants.
“of the mechanically ventilated schools only two contained more than 26 micro-organisms per litre, whereas of the naturally ventilated schools only three contained less than 26 per litre.”(Note that there were a total of 18 mechanically ventilated schools and 28 naturally ventilated schools investigated for airborne microbes.)“The all-important argument for mechanical ventilation is that it maintains a certain standard of purity, and, unless some simpler method which will maintain a similar standard can be devised, its adoption in crowded schools seems to be very much required.”	Building operation (including the source and rate of ventilation air delivery) impacts indoor microbes. This is consistent with finding x above (although these quantities of microbes are now known to be vastly underestimated).(Note that in this case “mechanically ventilated” meant that the schools were operated with dedicated outdoor air supply via mechanical means, and “naturally ventilated” meant that the schools were operated without mechanical means and primarily relied on infiltration [rather than open windows, despite the somewhat confusing use of a term that now refers to open-window ventilation]. In other words, the mechanically ventilated schools had higher ventilation rates [this was clear because carbonic acid concentrations {i.e., CO_2_ levels}] were higher in the naturally ventilated schools.)
“No constant relation between the quantities of carbonic acid, organic matter, and micro-organisms can be detected in individual cases.”“Sometimes we find a high organic matter accompanies by a low carbonic acid, whilst under other circumstances the reverse may be the case. A determination of carbonic acid alone is therefore never a sufficient indication of the purity or otherwise of a given sample of air.”	The concentration of indoor microbes and built environment parameters are weakly correlated, if at all. This is consistent with finding xi above.

Remarkably, many of the findings from Carnelley et al. (75) listed in Table 1 are consistent with some of the overarching findings from the more-recent studies of the microbiomes of indoor environments reviewed above. In fact, 7 of these 12 enumerated findings from more-recent investigations using molecular methods (specifically findings iii, v, vi, viii, ix, x, and xi) were identified or suggested in the 1887 paper, albeit in an admittedly broad and somewhat crude manner, and without much complexity. The following >100 years of the use of culture-based methods to sample microbes in buildings continued to build on many of these same themes, but this new body of work in the last 10 to 15 years using modern molecular methods has very rapidly added much needed nuance and complexity to our understanding of indoor microbiomes. However, it remains to be seen how this new knowledge base will change how we design, build, and operate buildings.

## SUMMARY

We are just now beginning to understand the complexity with which indoor microbiomes may affect human health in both positive and negative ways, but much more research is needed to better understand these complicated interactions. Although the use of molecular methods to analyze microbial samples has greatly increased the complexity with which we understand indoor microbes, we still know much more about relatively simple microbial characterizations based on sequence information (e.g., relative abundance of certain taxa and overall measures of diversity and richness) than we do about the function, expression, and viability of the vast numbers of microorganisms present inside buildings. Until molecular methods and statistical techniques advance to a state in which more-complex microbial characteristics (e.g., gene expression and function) can easily and cheaply be assessed in environmental samples, the true usefulness of molecular techniques may be best realized when used in conjunction with traditional methods of culturing and viability assessments (15). In fact, looking back at one of the earliest applications of molecular methods to indoor environments, in 2007, Lee et al. stated that “the combination of culture and culture-independent methods provided powerful means for determining both viability and diversity of bacteria in child-care facilities” (10). I would tend to agree.
